# A mouse model of vitiligo based on endogenous auto-reactive CD8 + T cell targeting skin melanocyte

**DOI:** 10.1186/s13619-022-00132-9

**Published:** 2022-10-02

**Authors:** Daoming Chen, Zijian Xu, Jun Cui, Ting Chen

**Affiliations:** 1 National Institute of Biological Sciences, Beijing, China; 2 Tsinghua Institute of Multidisciplinary Biomedical Research, Tsinghua University, Beijing, China

**Keywords:** Vitiligo, Mouse model, Activation, Endogenous autoreactive cytotoxic CD8 + T cells

## Abstract

Vitiligo is the most common human skin depigmenting disorder. It is mediated by endogenous autoreactive CD8 + T cells that destruct skin melanocytes. This disease has an estimated prevalence of 1% of the global population and currently has no cure. Animal models are indispensable tools for understanding vitiligo pathogenesis and for developing new therapies. Here, we describe a vitiligo mouse model which recapitulates key clinical features of vitiligo, including epidermis depigmentation, CD8 + T cell infiltration in skin, and melanocyte loss. To activate endogenous autoreactive cytotoxic CD8 + T cells targeting melanocytes, this model relies on transient inoculation of B16F10 melanoma cells and depletion of CD4 + regulatory T cells. At cellular level, epidermal CD8 + T cell infiltration and melanocyte loss start as early as Day 19 after treatment. Visually apparent epidermis depigmentation occurs 2 months later. This protocol can efficiently induce vitiligo in any C57BL/6 background mouse strain, using only commercially available reagents. This enables researchers to carry out in-depth in vivo vitiligo studies utilizing mouse genetics tools, and provides a powerful platform for drug discovery.

## Background

Vitiligo is a common skin autoimmune disease that affects about 1% of the world’s population (Kruger and Schallreuter 2012; Sehgal and Srivastava 2007; Silverberg 2015). It is caused by auto-reactive CD8 + T cells targeting pigment-producing melanocytes thus giving rise to skin depigmentation, which is usually presented as white spots and patches with distinct margins (Ezzedine et al. 2012). Although vitiligo is not life-threatening, social stigma and low self-esteem associated with the skin lesions have severe negative impacts on patients’ lives, in areas ranging from employment, relationships to mental health (Hamidizadeh et al., 2020).

Previous studies have shown that, compared with the healthy control group, the number of autoreactive CD8 + T cells is increased in both peripheral blood and skin of patients with vitiligo (Boniface et al. 2018; Mantovani et al. 2002; Ogg et al. 1998; Palermo et al. 2001; Van den Boorn et al. 2009). Our own histological analysis revealed extensive CD8 + T lymphocytes infiltration at the border of depigmented lesions (Xu et al. 2022). Vitiligo specific CD8 + T cells recognize multiple different melanocyte-specific antigens, including MART-1_26–35_, TYR_369-377_, gp100_154-162_, gp100_209-217_, and gp100_280-288_ (Boniface et al. 2018; Mantovani et al. 2002; Ogg et al. 1998; Palermo et al. 2001; Van den Boorn et al. 2009). Further studies revealed these melanocyte-specific T cells from lesion and perilesional skin could induce apoptosis in non-lesional autologous melanocytes ex vivo (Van den Boorn et al. 2009; Wu et al. 2013), and lysis of melanoma cells in vitro (Mantovani et al. 2002; Palermo et al. 2001; Wankowicz-Kalinska et al. 2003). These studies demonstrate the pathological role of endogenous auto-reactive CD8 + T cells in human vitiligo.

As a common skin autoimmune disease that is easy to diagnose but difficult to treat or cure, vitiligo has urgent medical need. In previous studies multiple mouse models of vitiligo have been developed to meet this need. Topical application of chemical reagent like monobenzone has been used to induce skin depigmentation in mice (Zhu et al. 2013). Skin depigmentation in this model is mainly caused by chemical stress induced melanocyte loss, which does not fully recapitulate the pathological features of auto-reactive CD8 + T cells caused melanocytes loss in vitiligo patients. More commonly used vitiligo mouse models rely on transgenic technology (Eby et al. 2014; Gregg et al. 2010; Miao et al. 2018; Mosenson et al. 2012) and adoptive transfer (Harris et al. 2012) of melanocyte antigen-recognizing transgenic CD8 + T cells. The transgenic CD8 + T cells in these models only target one specific melanocyte antigen, which is different from the diverse antigen-specific CD8 + T cells found in vitiligo patients. In addition, the adoptive transfer-based vitiligo induction method is technically complex, which limits its application in carrying out in-depth mechanistic studies using additional mouse genetic tools. Previous studies have also shown a melanoma mouse model with CD4 + T cell depletion developed hair coat depigmentation (Byrne et al. 2011; Malik et al. 2017; Zhang et al. 2007). In these studies, only hair coat depigmentation at the tumor dissection site was analyzed, which was believed to be a side effect of the anti-tumor immunity and different from the epidermis depigmentation occurred in most vitiligo patients.

In vitiligo patients, Tregs were found to be significantly decreased in both peripheral blood and skin (Dwivedi et al. 2013; Hegab and Attia, 2015; Klarquist et al. 2010). This was proposed to be a main mechanism leading to endogenous autoreactive CD8 + T cells escape anergy and induce vitiligo in patients (Maeda et al. 2014). Consistently, replenishment of Tregs stopped skin depigmentation in vitiligo mice model (Chatterjee et al. 2014; Eby et al. 2015). Clinical studies have reported that 13.5–25.7% of melanoma patients spontaneously develop vitiligo after receiving checkpoint inhibitor therapy, which is associated with a higher rate of objective response and patient survival (Failla et al. 2019; Herzberg and Fisher, 2016; Hua et al. 2016; Nardin et al. 2020). The immunotherapy induced vitiligo mainly results from activated endogenous anti-tumor CD8 + T cells recognizing antigens shared by melanoma cells and melanocytes (Lo et al. 2021). The percentage of CD8 + T cells specific for melanocyte antigens, including MART-1_26–35_, TYR_369-377_, gp100_209-217,_ and gp100_280-288_, were significantly increased in immune therapy responders (Lo et al. 2021; Palermo et al. 2005; Tjin et al. 2011). Interestingly, patients with vitiligo were also reported to be protected from developing melanoma (Paradisi et al. 2014). These studies indicate the endogenous auto-reactive CD8 + T cells in melanoma-related vitiligo share similarities with CD8 + T cells in conventional vitiligo.

Based on these clinical studies, here we describe a vitiligo mouse model by activating endogenous auto-reactive CD8 + T cells to target epidermal melanocytes. The strategy consists of brief inoculation of B16F10 melanoma cells and CD4 + regulatory T cell depletion. In human skin melanocytes are located in both epidermis and hair follicle; in mouse skin melanocytes are only present in hair follicles in dorsal skin, but are present in both epidermis and hair follicle in tail skin. To study the key feature of epidermis depigmentation and epidermal melanocyte loss in patients, we used mouse tail skin in this vitiligo mouse model. Since tail skin is physically separate from the tumor dissection site in dorsal region, our analysis also avoids the influence of tumor dissection and anti-tumor immunity in dorsal skin area. In previous vitiligo studies, quantification of vitiligo severity was often carried out by estimation of epidermis depigmentation level. To quantify at cellular level the progression and difference of vitiligo, we not only used FACS but also pioneered a whole-mount immunofluorescent staining strategy to visualize and quantify CD8 + T cell infiltration and melanocyte loss in tail skin epidermis. In our recently published work, we carried out systemic analyses of this mouse vitiligo model, and found it recapitulates all the hallmarks of human vitiligo disease at phenotypic, histological and signaling levels (Xu et al. 2022).

Fibroblasts play an essential role in skin homeostasis, wound healing, and immunity (Driskell and Watt, 2015). Our previous work has revealed that IFN-γ responsive fibroblasts are required to regulate endogenous auto-reactive CD8 + T cell local recruitment and drive patterned skin autoimmunity during vitiligo progression (Xu et al. 2022). Thus, the role of fibroblast in regulating immune cell activity in other skin diseases needs to be further investigated. Here, we also introduce an in vivo assay utilizing lentivirus-mediated shRNA knock-down in mouse skin fibroblasts to investigate the function of fibroblasts in skin homeostasis and immunity (Xu et al. 2022; Yu et al. 2018).

For complete details on the use and execution of this protocol, please refer to Xu et al. (2022).

## Methods

### Experimental design

**Fig. 1:**
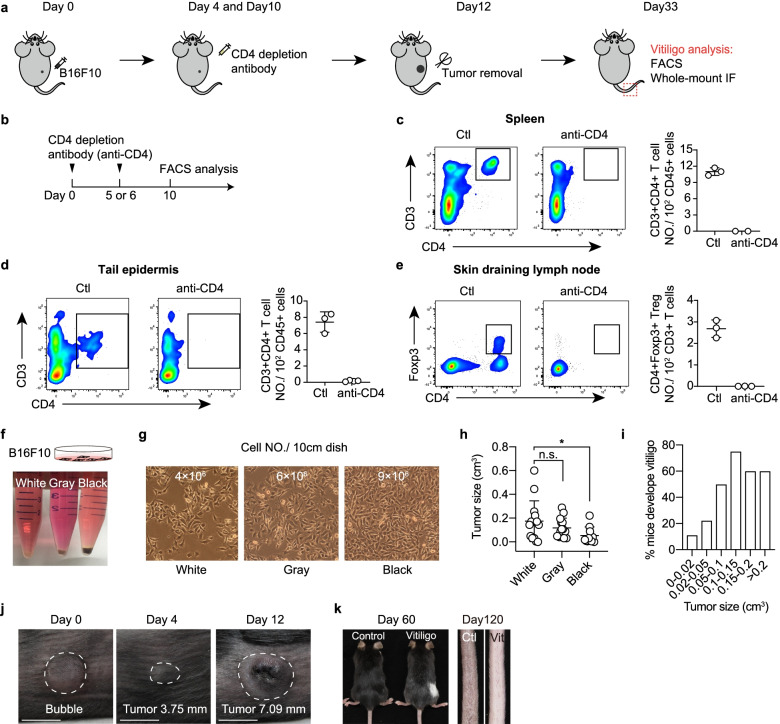
Schema for vitiligo mouse model induction. **a** Schematic diagram of the induction procedure for vitiligo mouse model. **b** Experimental procedure to evaluate the depletion efficiency of anti-CD4 depletion antibody. Two doses of anti-mouse CD4 depletion antibody were injected intraperitoneally, at 10 μg/g body weight (~ 200 μg) per animal per day. (**c**-**d**) Representative FACS profiles and quantification of CD3 + CD4 + T cells in spleen **c**, and tail skin epidermis **d**, of mice that were either untreated (Ctl) or treated with anti-CD4 depletion antibody. Cells were pre-gated on CD45 + live singlets. **e** Representative FACS profiles and quantification of CD4 + Foxp3 + Tregs in skin draining lymph node with or without anti-CD4 depletion antibody treatment. Cells were pre-gated on CD45 + CD3 + live singlets. **f** Representative images to illustrate the color of cell pellets of B16F10 cells under different culture conditions. **g** Representative brightfield images in a petri dish and cell counts of B16F10 cells that will yield cell pellets with different colors. **h** The tumorigenesis capacity of B16F10 cells with different colors of pellets. The tumor size was measured at Day 12 post B16F10 inoculation. **i** Quantifications represent the percentage of vitiligo developed (determined by marked melanocyte loss and CD8 + T cell infiltration at Day 33) with different tumor size at Day 12 post B16F10 inoculation. *n* = 10 (0–0.02 cm^3^), 9 (0.02–0.05 cm^3^), 6 (0.05–0.1 cm^3^), 8 (0.1–0.15 cm^3^), 5 (0.15–0.2 cm^3^), and 6 (> 0.2 cm^3^). **j** Photographs of injection area at Day 0, 4, and 12 of B16F10 intradermal injection. A small bubble will appear underneath the skin after the injection procedure. The tumor size is 2–4 mm on Day 4 and 5–15 mm on Day 12 in diameter. **k** Representative images of dorsal hair coat on Day 60 and tail on Day 120 after the vitiligo induction

**Fig. 2:**
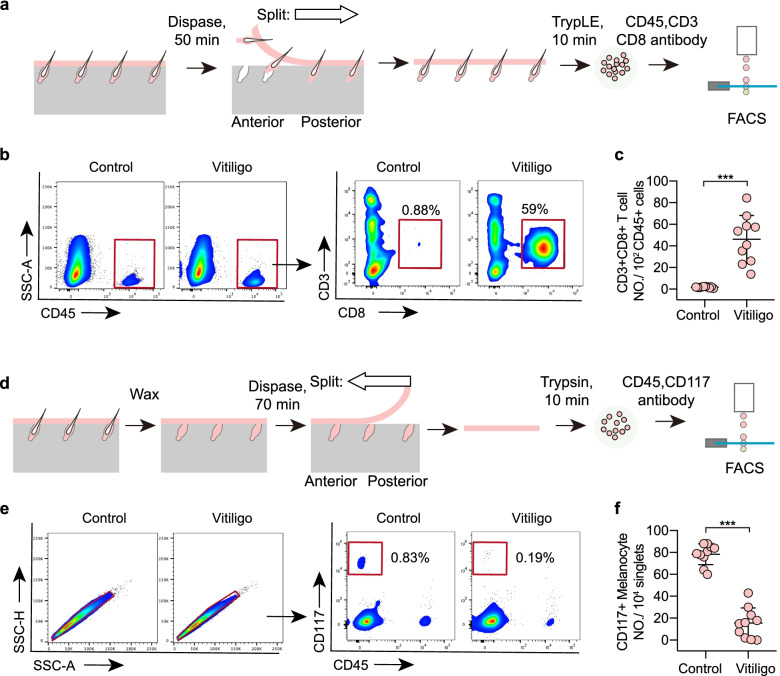
FACS to evaluate vitiligo induction efficiency. **a** Schematic diagram of the protocol for FACS analysis of CD3 + CD8 + T cells in tail skin epidermis. **b**-**c** Representative FACS profiles and quantification of CD3 + CD8 + T cells (pre-gated on CD45 + live singlets) on Day 33 after vitiligo induction. **d** Schematic diagram of the protocol for FACS analysis of CD117 + melanocytes in tail skin epidermis. **e**–**f** Representative FACS profiles and quantification of CD117 + melanocytes (pre-gated on live singlets) on Day 33 after vitiligo induction

**Fig. 3:**
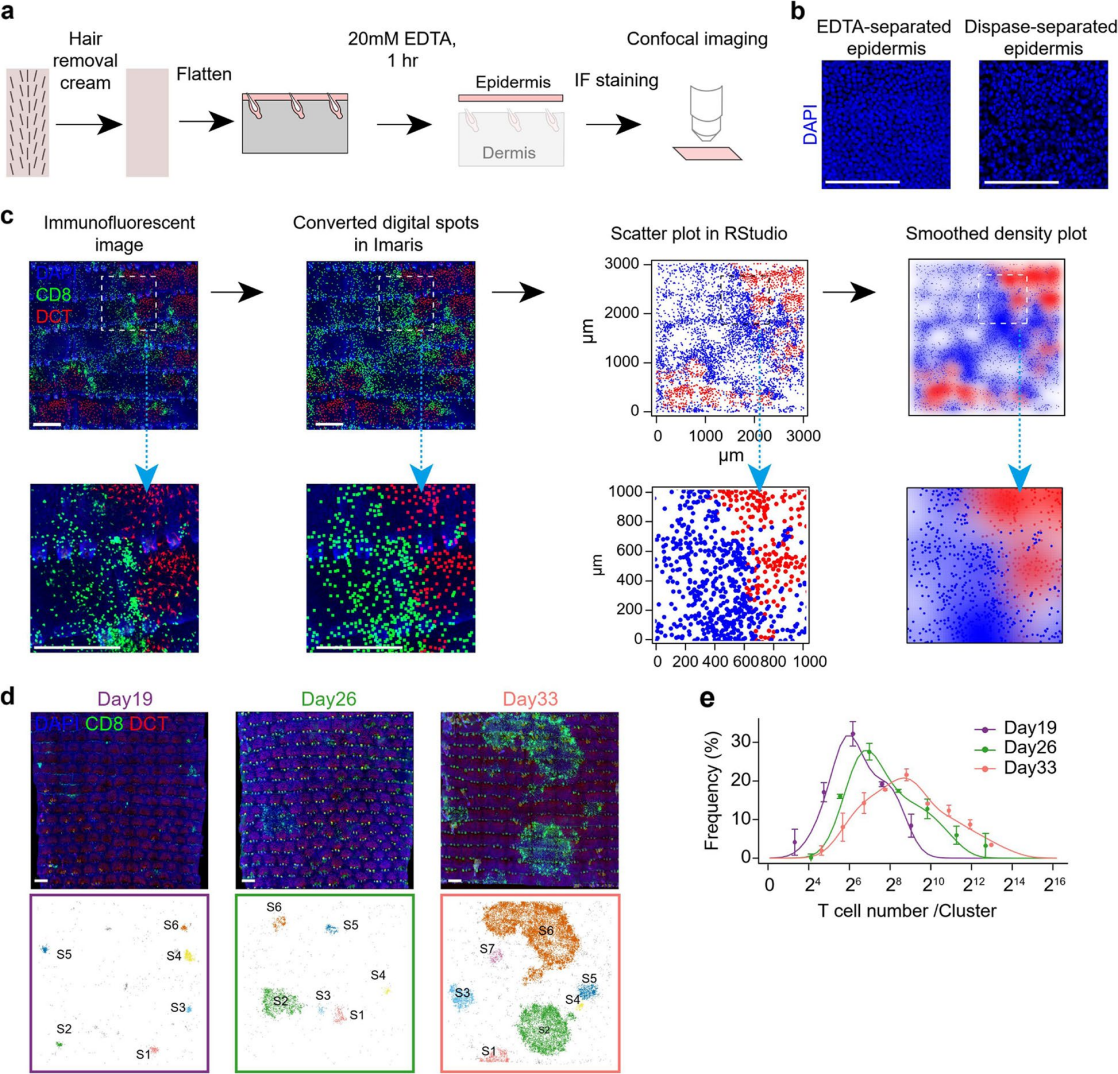
Whole-mount immunofluorescence staining to evaluate vitiligo induction efficiency. **a** Schematic diagram of the protocol for whole-mount immunofluorescent staining to detect melanocytes and CD8 + T cells in tail skin epidermis. **b** Representative whole-mount immunofluorescent images of epidermis separated by 20 mM EDTA or 1 × dispase. Scale bars, 100 µm. **c** Representative whole-mount immunofluorescent images, converted digital spots, scatter plots, and smoothed density plots showing the distribution pattern of melanocytes and CD8 + T cells on Day 33 after vitiligo induction. Scale bars, 500 µm. **d** Top: Representative whole-mount immunofluorescent images of melanocytes and CD8 + T cells on Day 19, 26, and 33 after vitiligo induction. Bottom: Scatter plots showing the distribution pattern of CD8 + T cells on the indicating time. Scale bars, 500 µm. **e** Quantification of T cell cluster size on Day 19, 26, and 33 after vitiligo induction. *n* = 8, 9, 7 mice for Day 19, 26, and 33, respectively

**Fig. 4:**
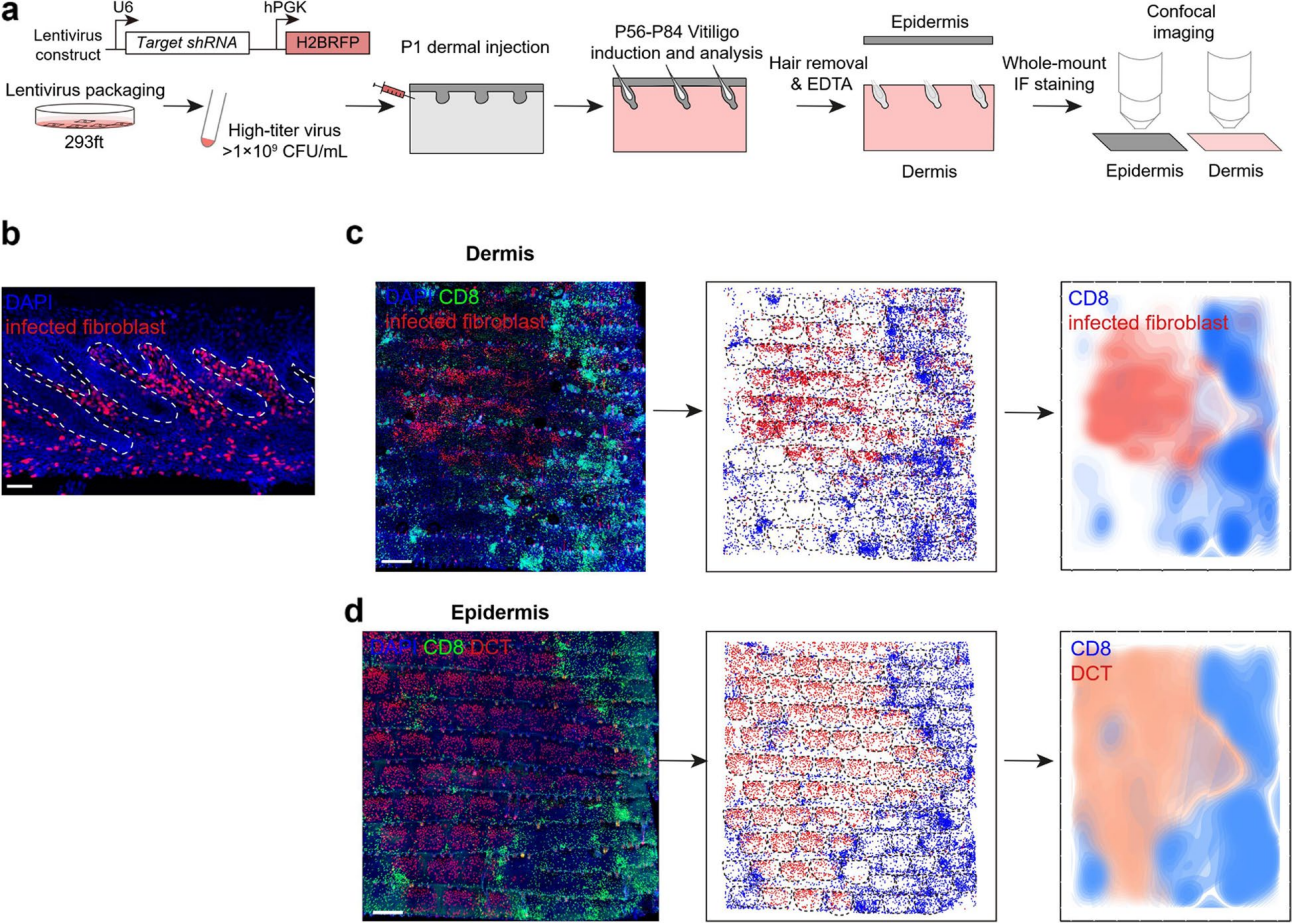
Lentivirus-mediated shRNA knockdown in skin dermal fibroblasts. **a** Schematic diagram of lentivirus-mediated shRNA knockdown in the skin dermal fibroblasts. **b** Representative image of lentivirus-injected area in skin section. Lentivirus infected cells are marked in red by lentivirus expressed H2B-RFP. Scale bar, 50 µm. **c**-**d** Whole-mount analysis of distribution pattern of dermal CD8 + T cells and infected fibroblasts (**c**), and epidermal CD8 + T cells and melanocytes (**d**) post *Stat1*-shRNA-containing lentiviruses injection and vitiligo induction. Black dotted line frames indicate the scale region of the tail epidermis. Scale bars, 500 µm

An overview of the experimental design is outlined in Fig. 1a. The procedure is comprised of three major parts shown in Figs. 1, 2 and 3: B16F10 cell preparation (Steps 1–16), vitiligo induction (Steps 17–28), and analysis using FACS (Steps 29–41) or whole-mount staining (Steps 42–53). For lentivirus-mediated functional studies of fibroblasts, the in vivo knockdown experiment is demonstrated in Fig. 4 (Steps 55–57).

In our laboratory, 60–80% of mice develop epidermal depigmentation using this protocol. The success rate was higher for female mice than for male mice. Therefore, we recommend that researchers prepare 10 female mice per group to ensure that at least 5 replicates will be obtained. Given that the B16F10 cell line was originally generated from melanoma in C57BL/6 mice (Poste et al. 1980), recipient mice must be syngeneic C57BL/6 or semi-syngeneic F1 mice.

### Materials

#### Mice

In this vitiligo induction protocol, we used 7- to 9-week-old female C57BL/6 mice, and at least 10 mice per experimental group are recommended. The age range of 7–9 weeks is optimal for vitiligo induction since the induction efficiency is reduced when using younger or older animals. The complete immune system is not fully developed in mice aged less than 5 weeks, thus these mice are not suitable to induce vitiligo. Mice aged 5–6 weeks normally have dorsal skin in anagen and the thickened skin makes intradermal injection of B16F10 difficult, which results in B16F10 leakage and impairs the induction efficiency. Mice in 7–9 weeks are used for vitiligo induction. The mouse hair cycle enters a resting phase at postnatal 7 weeks, and lasts for at least 6 weeks. At this stage, the skin is thinnest and intradermal injections go smoothly without leakage. Though dorsal skin is in the telogen stage on the day B16F10 is transplanted, the mice aged 10–12 weeks will enter anagen during the one-month vitiligo induction procedure. It is speculated that the low efficiency of vitiligo induction is due to immunosuppressive molecules generated which provide immune privilege in anagen. After 12 weeks, the entry into anagen become sporadic and asynchronized, making it harder to predict skin thickness and conduct intradermal injections. And for the purpose to save time and feeding cost, we do not recommend using mice older than 10 weeks.

For genetic manipulations of dermal fibroblasts, lentivirus was injected into the tail of newborn C57BL/6 mice, and CD1 nursing surrogate mothers were used to foster newborn mice.

All animals are maintained in a specific pathogen-free (SPF) facility.

#### Reagents

##### Cell line

- B16F10 cells: ATCC, Cat# CRL-6475

##### Antibodies

- InVivoMAb anti-mouse CD4 depletion antibody: Bio X Cell, Cat# BE0003-1
- FITC Rat anti-mouse CD45: BioLegend, Cat# 103,108 (1:300)
- APC Rat anti-mouse CD8a: Thermo Fisher Scientific, Cat# 17–0081-82 (1:300)
- PE/Cyanine7 Rat anti-mouse CD3: BioLegend, Cat# 100,220 (1:300)
- PE Rat anti-mouse CD117: BD Biosciences, Cat# 553,355 (1:300)
- APC anti-mouse CD4: BioLegend, Cat# 100,412 (1:300)
- PE anti-FOXP3: Thermo Fisher Scientific, Cat# 12–5773-82 (1:300)
- Foxp3 / Transcription factor staining buffer set: Thermo Fisher Scientific, Cat# 00–5523-00
- Rabbit anti-mouse Dct: Chenting Lab made (1:3000)
- Alexa Fluor 488 Donkey Anti-Rabbit IgG: Jackson ImmunoResearch, Cat# 711–545-152 (1:1000)
- Alexa Fluor 647 Donkey Anti-Rat IgG: Jackson ImmunoResearch, Cat# 712–605-153 (1:1000)

##### Chemicals

- Dulbecco's modified Eagle medium (DMEM): Thermo Fisher Scientific, Cat# C11965500
- Fetal bovine serum (FBS): Thermo Fisher Scientific, Cat# 10099141C
- Penicillin–streptomycin (10,000 U/mL): Thermo Fisher Scientific, Cat# 15,140–122
- DPBS: Thermo Fisher Scientific, Cat# 14,190,144
- Trypsin–EDTA (0.25%), phenol red: Thermo Fisher Scientific, Cat# 25,200,056
- Trypan blue solution, 0.4%: Thermo Fisher Scientific, Cat# 15,250,061
- Isoflurane: RWD, Cat# R510-22–16
- Hair removal cream: Veet
- Hair removal wax strips: Veet
- Dispase II, powder: Thermo Fisher Scientific, Cat# 17,105,041
- TrypLE express enzyme (1X), phenol red: Thermo Fisher Scientific, Cat# 12,605,010
- Ethylenediaminetetraacetic acid (EDTA), disodium salt, dihydrate: Sinopharm, Cat# 10,009,717
- Paraformaldehyde: Sigma-Aldrich, Cat# 158,127
- TritonX-100: Sigma-Aldrich, Cat# X100
- Bovine serum albumin: Sigma-Aldrich, Cat# V900933
- Normal donkey serum: Jackson ImmunoResearch, Cat# 017–000-121
- DAPI: Sangon, Cat# D6584
- Glycerol: Sigma-Aldrich, Cat# G5516

#### Equipment

- Cell culture incubator with 5% CO_2_ humidified air: Thermo Fisher Scientific, Cat# HERACELL150i
- Centrifuge: Beckman, Cat# Allegra X-15R
- Automated cell counter: Cellometer, Cat# K2
- Animal anesthesia system containing an air pump, a small anesthesia machine, an induction chamber, anesthesia tubing masks, and a gas filter canister: RWD
- Electric razor
- Handheld vacuums for animal hair
- 1 mL insulin syringe: BD Biosciences, Cat# 328,421
- 1 mL syringe: BD Biosciences, Cat# 300,841
- Sterile scissors
- Fine-tipped tweezers
- Reflex skin closure system to suture wounds
- Flow cytometer: BD Biosciences, FACS Aria™ III or Fusion
- Orbital shaker: Kylin-bell lab instruments, Cat# TS-1
- Confocal microscope: Nikon, Cat# A1R MP

#### Software

- Flowjo: FlowJo LLC, FlowJo™ 10.4
- GraphPad Prism 8
- Imaris: Bitplane, Imaris 7.4.2
- R
- RStudio

#### Reagent and equipment setup

- **Anti-CD4 depletion antibody solution** Aliquot anti-CD4 depletion antibody with 1.2 mg per tube according to the actual antibody concentration, and then freeze at -80˚C. Avoid repeated freeze and thaw cycles. On the day of use, thaw an aliquot and dilute to 1 mg/mL with sterile DPBS. Upon receiving a new antibody lot, we recommend testing the depletion efficiency of CD4 + T cells and regulatory T cells (Fig. 1b-e). We used FACS to quantify CD45 + CD3 + CD4 + Foxp3 + Treg cells in spleen, tail skin epidermis and skin draining lymph node to validate efficient and complete depletion of Treg cells after anti-CD4 depletion antibody administration.
- **B16F10 culture medium** Add 50 mL FBS and 5 mL Pen-strep to 445 mL DMEM. Aliquot in 50 mL tubes and use within one week.
- **B16F10 freezing medium** Prepare a mixture of 50% growth medium with 40% FBS and 10% DMSO, filter sterilize prior to use.
- **5% FBS** Dilute 5 mL FBS with 95 mL DPBS to a final concentration of 5%. Prepare fresh solution on the day of use.
- **1** × **dispase working solution** Dissolve 13.3 mg dispase (1.8 U/mg) in 10 ml DPBS to make a 2.4 U/mL working solution. Prepare fresh solution on the day of use.
- **4% PFA (pH 7.4)** Add 40 g paraformaldehyde to 800 mL of DPBS. Heat while stirring to approximately 60 °C. Slowly raise the pH by adding NaOH dropwise from a pipette until the solution clears. Afterward, the solution should be cooled and filtered. Adjust the pH of the solution to pH 7.4 with HCl. Adjust the volume of the solution to 1 L with DPBS. The solution can be aliquoted and stored at 2–8 °C for a week or frozen at -20 °C for up to 12 months.
- **20 mM EDTA (pH 8.0)** Dissolve 7.3 g EDTA in 50 mL DPBS to make a 0.5 M stock solution and adjust the pH to 8.0. Dilute it with 1 × DPBS at 1:25 to give a 20 mM working solution. Store at room temperature.
- **Blocking buffer** Dissolve 0.25 g BSA, 0.5 mL normal donkey serum, and 75 μL TritonX-100 in 25 mL DPBS. Store at -20˚C in 5 mL aliquots for up to 12 months.
- **50% glycerol** Dilute 25 mL glycerol with 25 mL DPBS to make a 50% glycerol solution. Store at room temperature.
- **Lentivirus** Produce and concentrate high titer (> 1 × 10^9^ CFU/mL) lentivirus as previously reported (Chen et al. 2012; Yu et al. 2018). Aliquot the high titer virus (10 μL/tube) then freeze at -80˚C. Avoid repeated freeze and thaw cycles. On the day of use, thaw an aliquot and place it on ice before injection.
- **Wide-mouth P1000 tips** Use scissors to cut ~ 5 mm off the end of a 1 mL pipette tip.

### Procedure

This experimental procedure describes how to conduct vitiligo induction with C57BL/6 mice and how to evaluate the vitiligo induction efficiency with FACS and/or whole-mount immunofluorescent (IF) staining. In addition, a protocol for lentivirus-mediated shRNA knockdown in skin dermal fibroblasts is described.

#### Preparation of B16F10 cells

##### Thaw B16F10

The B16F10 cell line is stored in liquid nitrogen with 1.5 × 10^6^ cells in 1 mL of freezing medium per vial.

1. Remove the frozen B16F10 cells from liquid nitrogen and thaw quickly in a 37˚C water bath.
2. Immediately after the cells are completely thawed, decontaminate the vial with 70% ethanol, and transfer the cells to a sterile 15 mL tube containing 10 mL B16F10 culture medium.
3. Centrifuge the B16F10 cells at 350 × *g* for 5 min, remove supernatant and resuspend the cells in 1 mL of culture medium.
4. Transfer the B16F10 cells to a 10 cm cell culture dish containing 10 mL of B16F10 culture medium.
5. Culture the B16F10 cells at 37˚C with 5% CO_2_ and monitor until the cells have reached 50–60% confluence.
6. Proceed to subculturing B16F10 cells.

**⚠CRITICAL:** We recommend subculturing B16F10 cells for 3-5 passages after thawing before use for the vitiligo induction.

##### Subculturing B16F10 cells

7. Remove all medium from the dish and wash the B16F10 cells once with 3 mL DPBS to remove excess medium and serum.
8. Add 3 mL of 0.25% Trypsin/EDTA solution to the monolayer and incubate for 3–4 min at 37˚C until cells detach. Check the cells under a microscope and confirm that all the cells have detached.

**⚠CRITICAL:** If cells are still attached, gently tap the sidewall of the culture dish until all the cells have detached.

9. Add 3 mL B16F10 medium and transfer the cell suspension to a 15 mL centrifuge tube.
10. Centrifuge the cells at 350 × *g* for 5 min, remove supernatant and resuspend the cells in 1 mL of culture medium.

Note: We have found that B16F10 cells which are gray-to-white in color when pelleted confer superior vitiligo induction potential (Fig. 1f-i).

11. Perform counts for both viable cells and total cells (see “Determine viable and total cell counts” in steps 14–16). In a 10 cm cell culture dish, the total number of cells should be 6 × 10^6^ at 80% cell confluence.

**⚠CRITICAL:** Never allow the B16F10 cells to reach more than 80% confluence during subculturing. Once a B16F10 culture approaches 80–100% confluence, both the culture medium and the cell pellet will turn black (Fig. 1g); such B16F10 cells display a dendritic morphology and have inferior melanoma and vitiligo induction potential (Fig. 1h-i). Do not use such cells and commence with a newly thawed vial of cells.

12. Seed 1.5 × 10^6^ cells in a 10 cm dish containing 8 mL of B16F10 medium.
13. Culture the cells at 37˚C with 5% CO_2_. 1.5 × 10^6^ cells will grow to 6 × 10^6^ in 24 h.

##### Determine viable and total cell counts

14. Following step 11, transfer 15 μL resuspended cells to a microcentrifuge tube and add 15 μL Trypan blue stain (0.4%) solution. Gently pipette to mix.
15. Count the total number of cells and viable cells using an automated cell counter. **⚠CRITICAL:** To avoid human-introduced variations, we recommend the use of automatic cell counters over hemocytometer chambers.

**⚠CRITICAL:** For the subsequent intradermal injection, the cell viability should be ≥95%.

16. For the intradermal injection protocol for vitiligo induction, at least 2 × 10^5^ B16F10 viable cells are needed per mouse.

Note: Considering the likely cell loss during the intermediate steps, prepare a 1.5-fold total cell number per experiment. For example, we prepare 3×10^6^ B16F10 viable cells for vitiligo induction in 10 mice.

#### Vitiligo induction

17. Adjust the B16F10 cell concentration to 1.6 × 10^3^/μL in the culture medium, which corresponds to the final 2 × 10^5^ cells B16F10 cell inoculum per 125 μL injection volume.

Note: Keep the cell suspension on ice before injection.

18. Anesthetize mice with isoflurane in an animal anesthetic system. Turn on the power of the air pump and anesthesia machine. We usually set the highest speed of airflow (~ 4L/min according to the machine manual) and set the anesthesia machine gear to 3 to make a 3% isoflurane flow. After the flow is established, put the mice into the induction chamber to anesthetize them. To make sure the mouse is fully anesthetized, one can gently rock the chamber side to side to see if the animal is still conscious.
19. Once the mice are fully anesthetized, remove the mouse from the induction chamber and place it on its back with its nose directly within the anesthesia tubing masks.

Note: Mice should not be anesthetized for more than 60 minutes.

20. Shave the right flank with an electric razor. Use a handheld vacuum above the mouse to remove shaved hairs.

**⚠CRITICAL:** Do not use the mice if the hair cycle enters into anagen (the skin color turns to gray or even black (Muller-Rover et al. 2001).

21. Use an insulin syringe to deliver 125 μL of the B16F10 cells to the right flank skin via intradermal injection. This day is recorded as Day 0.

Note: Before loading B16F10 cells into the insulin syringe, flick cube to mix.

**⚠CRITICAL:** The angle of intradermal injection should be 5 to 15 degrees, slightly bend the needle of the syringe to make the injection angel almost parallel to the skin surface, with the bevel side up. The needle is inserted to a depth of 3 mm (1/8 inch), with the entire bevel inside. Slowly inject the solution, a small bleb should appear within the skin (Fig. 1j).

22. On Day 4, check the tumor size with a vernier caliper at the intradermal injection site. Most tumors are 2–4 mm in diameter at this time point (Fig. 1j).

**⚠CRITICAL:** In general, more than 90% of mice will develop tumors that exceed 2 mm in diameter. A rate below 90% likely indicates problems with the viability or number of B16F10 cells, and/or problems with the intradermal injection procedure.

23. On Day 4, weigh the mice using an electronic scale, and calculate the dose of CD4 depletion antibody (10 μg/g mouse weight) to administer.

Note: On the day of use, thaw an aliquot and dilute to 1 μg/μL with sterile DPBS. Considering the likely antibody loss during the intermediate stage, make a 1.2-fold preparation for the injection antibody.

24. Use a 1 mL syringe to deliver the CD4 depletion antibody via intraperitoneal injection.

Note: After injecting the CD4 depletion antibody, retract the needle slowly.

25. On Day 10, weigh the mice again, and calculate the dose of CD4 depletion antibody (10 μg/g mouse weight) to administer.

Note: Over these six days, the mice will gain 1-3 g of body weight.

26. Repeat the injection of the CD4 depletion antibody as in Step 24.
27. On Day 12, surgically resect the melanoma from the right flank. Typically, at this stage the tumor diameter is between 5–15 mm (Fig. 1j). Anesthetize the mouse and keep it anesthetized as in Steps 18–19. Shave the hair near the melanoma with an electric razor. Incise the skin at the base of the tumor. Insert the tips of sterile scissors into the incision site and gently spread to the adjacent region, developing a dissection plane between the nearby skin and the tumor. Extend the incision to expose the tumor (if needed), and resect the tumor from the adherent deep tissue.

Note: Skin above the tumor should be resected with the B16F10 tumor.

**⚠CRITICAL:** Be careful not to cut through the tumor during surgery. Try to completely resect the tumor, for the residual melanoma cells will result in recurrent primary tumors.

28. Use suture clips to close the wounds. After closure, clean the surgical area with gauze. We recommend placing the mouse on a heating pad for at least 15 min until it regains consciousness.

Note: At Day 60 hair follicle close to the tumor dissection site will appear white while the rest of the dorsal skin hair follicle is still black (Fig. 1k). Hair follicles in 8- to 9-week-old female C57BL/6 mice dorsal skin are in telogen, but new hair shaft formation is induced by wounding close to the tumor dissection site. In tail skin, complete epidermis depigmentation is normally visible at Day 120 after induction, although at cellular level melanocytes loss is much earlier than this time point. This is due to the fact that visible epidermis pigmentation results from keratinocytes up taking melanin from melanocytes, while complete keratinocytes turn over takes 1-2 month.

#### FACS and whole-mount immunofluorescent staining to evaluate vitiligo induction efficiency

On Day 33 after vitiligo induction, the efficiency of vitiligo induction can be evaluated by FACS (Fig. 2) and whole-mount IF staining (Fig. 3), based on quantification of epidermal melanocytes and CD8 + T cells.

29. Euthanize the mouse by CO_2_ exposure in a closed chamber at a flow rate of 3,000–4,000 mL/min for 6 min, then confirm the euthanasia by death signs such as arrested breathing and dilated pupils. Tail skin of the same mouse is used for both whole-mount staining and FACS analysis. But since they require different treatments, the skin is divided as following: use the middle 1/3 section of the tail skin (~ 3 cm), divide the section into 3 equal pieces along the vertical body axis (~ 1 cm each); one piece is used for whole-mount staining and should be processed as described in step 42; the other two pieces are processed differently for FACS analysis to quantify epidermal CD8 + T cells (step 30) and melanocytes (step 35) separately.

Note: The experiment follows the National Institute of Biological Sciences, Beijing guideline for the euthanasia method.

##### Analysis of CD8 + T cells using FACS (Fig. 2a-c)

30. Use scissors to cut off the skin along the vertical body axis from the ventral side and remove the skin segment for FACS analysis of epidermal CD8 + T cells from the tail vertebrae. Flatten the skin square in a 10 cm dish for at least 5 min before enzymatic digestion in the next step.

**⚠CRITICAL:** Do not wet the skin with water or DPBS. Cover the dish with a lid and place it on ice to prevent the skin from drying out.

31. Digest the skin with 2 mL 1 × dispase working solution in one well of a 24-well plate at 37˚C, with shaking at 80 rpm for 50 min.

Note: Treatment with dispase for more than 1 hour will impair surface antigen CD8a.

**⚠CRITICAL:** The skin should float dermis side down in the dispase, without any curling at the edges.

32. Separate the epidermis from the dermis using a fine-tipped tweezer in an anterior-to-posterior direction. Transfer the epidermis to one well of a 24-well plate containing 1 mL TrypLE. Incubate at 37˚C with shaking at 80 rpm for 10 min.

Note: Trypsin treatment will impair CD3 and CD8 antigen on cell surface. The epidermis should float with the inner side down in the solution, without any curling at the edges.

Note: For time-saving purposes, we usually keep the hair follicles on the epidermis when analyzing CD8+ T cells. On Day 33 after vitiligo induction, we did not detect infiltration of CD8+ T cells in tail skin hair follicles by whole-mount IF staining.

33. To stop digestion, add 1 mL cold 5% FBS to the well. Pipette the epidermis 20 times with wide-mouth P1000 tips to obtain a single-cell suspension.

Note: The suspension will become cloudy when the cells are sufficiently dissociated from the epidermis.

34. Filter the cells with a strainer (40 μm) and centrifuge at 450 × *g* for 5 min. Resuspend the cells in 300 μL 5% FBS, and stain for 15 min with CD45-FITC, CD3-PE-Cy7, and CD8-APC on ice, followed by washing once with 5% FBS.

**⚠CRITICAL:** One 1×1 cm^2^ tail skin sample normally generates ~10^6^ live cells. Record 1,000 events in CD45+ gates for each sample by FACS.

##### Analysis of melanocytes using FACS (Fig.  2d-f)

35. Use scissors to cut off the skin along the vertical body axis from the ventral side and remove the skin segment for FACS analysis of epidermal melanocytes from the tail vertebrae. Flatten the skin square in a 10 cm dish.
36. Prior to dispase digestion, wax strips are used to remove the hair shafts from the flattened tail skin. Press the wax paper onto the epidermis side, and then tear off the wax paper quickly in a posterior-to-anterior direction.

**⚠CRITICAL:** To quantify epidermal melanocytes, it is necessary to remove hair follicles from the epidermis because hair follicles also contain melanocytes. We find removing hair shaft facilitates separation of hair follicle from epidermis in the subsequent enzymatic treatment.

37. Digest the skin with 1 × dispase at 37˚C, with shaking at 80 rpm for 70 min.

**⚠CRITICAL:** This step requires 70 minutes of digestion to retain most hair follicles in the dermis.

38. Peel the epidermis from the dermis along the posterior-anterior direction with a fine-tipped tweezer swiftly. Use a fine-tipped tweezer to remove the occasional remaining hair follicles attached to epidermis under a dissection microscope.

**⚠CRITICAL:** During this step, the epidermis is peeled in the opposite direction to the procedure in Step 32 to get rid of hair follicles.

39. Transfer the epidermis to one well of a 24-well plate containing 1 mL 0.25% Trypsin medium. Incubate the plate at 37˚C, with shaking at 80 rpm for 10 min.
40. Obtain the single-cell suspensions as in Step 33.

Note: As hair follicles are retained in the dermis, this cell suspension will not be as cloudy as that obtained during step 33.

41. Filter the cells with a strainer (40 μm) and centrifuge at 450 × *g* for 5 min. Resuspend the cells in 300 μL 5% FBS, and stain for 15 min with CD45-FITC and CD117-PE, followed by washing once with 5% FBS. Record 20,000 events in the last singlets + gates for each sample by FACS.

Note: In Fig. 2, we illustrate how to quantify the percentage of CD8 + T cells and CD117 + melanocytes using FACS analysis. For absolute cell number quantification within fixed skin area, one can follow step 52 using whole-mount IF staining, or follow the method here: Measure the area of skin biopsy before enzymatic digestion (*A* mm^2^). Resuspend the obtained single cells in X volume of FACS buffer (*X* μL), then load Y volume of the suspension (*Y* μL) for flow cytometry and record the total number of cells of interest (*b* in total). The absolute number of cells per unit of area is calculated by the formula: $n (No./m{m}^{2})=\frac{Xb}{YA}$.

##### Analysis of melanocytes and CD8 + T cells using whole-mount staining (Fig. 3)

42. As described in step 29, about 1 × 1 cm^2^ tail skin biopsy is used for whole-mount staining. Hair shafts will interfere with visualization of epidermal staining signals. Before removing the skin segment from the tail vertebrae, apply hair removal cream to the surface of the tail and incubate at room temperature for 6 min.

Note: Incubation longer than 6 min will impair the epidermis and cause skin wound.

43. Gently and completely wipe off the hair removal cream with a damp tissue. Remove the processed tail skin from tail vertebrae.
44. Transfer the skin to a 5 mL tube containing 4 mL 20 mM EDTA, and incubate at 37˚C with shaking at 80 rpm for 1 h.

Note: The tube should lie flat on the shaker. Longer incubation will increase the number of hair follicles remaining in epidermis. Note for whole-mount analysis, we use EDTA instead of dispase to dissociate epidermis from dermis here, because we found that dispase treatment causes loosening of the epidermal cells connections, as indicated by increased nuclei distances among cells (Fig. 3b). For FACS analysis, we use diapase instead of EDTA to dissociate epidermis from dermis, because EDTA treatment leads to decreased cell viability compared to diapase treatment found in FACS analysis.

45. Separate the epidermis from the dermis. Use a fine-tipped tweezer to quickly peel the epidermis from the dermis in a posterior-to-anterior direction. Use a fine-tipped tweezer to remove the remaining hair follicles from the epidermis under a dissection microscope.

Note: Any remaining hair follicles will interfere with IF staining signals in the epidermis.

**⚠CRITICAL:** Both the quick peel action and the posterior-to-anterior direction are essential aspects for the successful removal of the hair follicles from the epidermis.

46. Prepare a 24-well plate that contains 1 mL DPBS in each well; place the epidermis into the well with the surface side facing up. Avoid any curling at the edges.
47. Aspirate DPBS off. Fix the whole-mount epidermis with 1.5 mL 4% paraformaldehyde (PFA) at room temperature for 10 min, wash 3 times with DPBS (15 min each), and then permeabilize in -20˚C methanol with 0.3% H_2_O_2_ at -20˚C for 20 min.
48. Block the whole-mount epidermis for 1 h in ~ 300 μL blocking buffer. Incubate samples with 300 μL blocking buffer containing 1 μg/mL DAPI and diluted primary antibodies: Rabbit anti-mouse Dct (1:3000) and Rat anti-mouse CD8a (1:300) overnight at 4 °C.
49. Wash samples with DPBS 3 times (15 min each). Incubate samples with 300 μL blocking buffer containing 1 μg/mL DAPI and diluted secondary antibodies: Donkey Anti-Rabbit Alexa Fluor 488 (1:1000) and Donkey Anti-Rat Alexa Fluor 647 (1:1000) for 1 h at room temperature.
50. Wash samples with DPBS 3 times (15 min each). Mount the tissue with 50% glycerol. Apply coverslips and seal the edges with a copious amount of clear nail polish. Store in dark at -20 °C.
51. Image the samples with a confocal microscope (*e.g.*, Nikon A1-R). The example images were acquired using a 20 × 0.75 objective lens for representative images (Fig. 3b) and a 10 × 0.5 objective lens for quantitative images (Fig. 3c). Z-stacks were acquired at a resolution of 1024 × 1024 for representative images and 512 × 512 for quantitative images. Microscopy data were analyzed using Imaris software with the 3D visualization module (Bitplane).
52. To quantify the number of melanocytes and CD8 + T cells, first, measure the width and height to calculate the area of the skin sample; second, convert the fluorescent signals to digital signals using the ‘Spot’ function; calculate the density of cells using the following formula: total number of spots/area.

Note: See Results and Conclusions for the absolute number per cm^2^ of CD8+ T cells and melanocytes.

53. To evaluate the distribution and density of CD8 + T cells and melanocytes, the position data coordinates of each digital spot were exported, edited, and then imported into RStudio. Create a scatter plot using the “ggplot2” package, then smooth the color density using the “smoothscatter” function (Fig. 3b) or create a heatmap using the “stat_density_2d” function (Fig. 4c and d).

#### Lentivirus-mediated shRNA knockdown in dermal fibroblasts

54. Prepare high titer shRNA-expressing lentiviruses (> 1 × 10^9^ CFU/mL) (Chen et al. 2012) Aliquot the high titer virus (10 μL/tube) then freeze at -80˚C. Thaw an aliquot on ice before injection.

Note: The knockdown efficiency should be evaluated *in vitro*. We used lentiviruses expressing H2BRFP to label cells expressing shRNA *in vivo*.

55. Use a BD insulin syringe to deliver 10 μL high titer lentivirus (> 1 × 10^9^ CFU/mL) into the tail skin of newborn P1 mice via intradermal injection.

Note: Cut specific toe to mark the injected pup, and clean the blood with gauze before returning the animal to its cage.

**⚠CRITICAL:** Load the virus into the base of the syringe with a 10μL pipette. To remove air from the syringe before injection, insert the syringe piston, and hold the syringe with the tip end facing upward: tap the syringe, then push the piston slowly upward. During injection, hold the mouse gently to prevent asphyxiation.

**⚠CRITICAL:** The needle needs to be inserted along the anterior-to-posterior direction at the tail base. The angle of the intradermal injection should be 5 to 15 degrees, with the tip opening side facing up. The needle should be inserted to a depth of 3 mm (1/8 inch), with the entire bevel inside. The skin will turn white once the virus has been injected. Slowly retract the needle after injection.

56. At 8–9 weeks after birth, induce vitiligo according to steps 1–28. On Day 33 after vitiligo induction, the function of shRNA targeted genes can be evaluated by whole-mount staining of both the epidermis and the dermis by analyzing the cell distribution of CD8 + T cells, melanocytes, and lentivirus targeted dermal fibroblasts.
57. Perform analysis according to steps 51–53. To quantify cell numbers per scale, import the whole-mount image and scatter plot in Adobe Illustrator, adjust the images to align digital spots and fluorescent signals, and draw the scale region according to the location of hair follicle triplets. Then select the spots in each scale, open Window > Document Info > Objects, and the number will be listed.

### Troubleshooting

**Table:** 

Step	Problem	Possible reason(s)	Possible solution(s)
10	The B16F10 pellet turns black	B16F10 cells approach more than 80% confluence before subculturing	Do not use this dish of cells and thaw a new vial
21	Difficulty in piercing the needle into the skin during B16F10 injection	The needle is blunt	Change to a new syringe after injecting 3 mice
21	Excessive leakage of B16F10 suspension from the injection site (> 1/3)	The hair cycle enters anagen and the skin thickens	Do not use such mice; use 8–9-week-old female mice with telogen skin
21	After injection, the emerged small bubble does not move with the skin when the skin is gently tugged	The needle is inserted subcutaneously or into the muscle layer	1. Insert the needle at an angle parallel to the skin surface (5–15°). Bending the needle slightly will help 2. Decrease the insertion depth of the needle
22	No tumor is detected on Day 4	1. B16F10 cells approach more than 80% confluence or turn black 2. The counts of B16F10 cells have not been correctly counted or calculated 3. The viability of B16F10 cells decreases after being kept on ice for more than 1 h 4. The viability of B16F10 cells can decrease upon transporting with a -20˚C ice pack	1. Ensure the B16F10 cells are in optimal status before use (< 80% confluence, and white-to-gray in color) 2. Determine the viable and total cell counts with an automated cell counter. Gently pipette to mix the cell suspensions before loading them onto the chip of a cell counter 3. Complete the B16F10 injection step within 1 h. Keep the cell suspension on ice before injection 4. Transport the B16F10 cells with ice
27	The diameter of the tumor is less than 5 mm on Day 12	B16F10 cells are heterogeneous in nature and previous publications have widely reported variable tumor growth after engraftment. Based on our experience, tumor with diameter less than 2 mm at Day 4, will not grow up to have diameter exceeding 5 mm on Day 12	1. We recommend that researchers prepare 10 female mice per group to ensure that at least 5 replicates will be obtained 2. Do not use mice with tumors smaller than 2 mm on Day 4
28	High incidence of death after tumor removal	1. The mice housing condition is not optimal 2. The suture clip on the wound is so loose that it fell off after operation 3. The tumor is so large that the wound is too large after the operation	1. Mice should be maintained in a specific pathogen-free facility with clean caging 2. Carefully affix the suture clip to sure closure 3. Strictly control the number of injected cells to less than 2 × 10^5^
33	Low yield of cells after enzyme and mechanical digestion	1. Dispase digestion or Trypsin/TrypLE digestion is not efficient 2. Mechanical digestion is not enough	1. Ensure enzyme solutions are freshly made. Dispase should be prepared on the day of use 2. Increase digestion time by 1–2 min 3. Make sure to repeatedly pipette the epidermis piece 20 times. An increase to 30 times may help
34	Cell viability was lower than 50% in FACS analysis	1. Excessive time for cell suspension preparation 2. Low yield of cells after enzyme and mechanical digestion	1. Complete the digestion and staining step within 3 h. If there are > 5 samples, we recommend having 2 people working together to minimize the digestion time 2. Refer to troubleshooting item 33
34	No positive staining of CD8 + markers by flow cytometry	Dispase digestion time exceeded 1 h. Longer dispase digestion impairs CD8 antigen	Shorten the dispase digestion time
34	No vitiligo development (No CD8 + T infiltration or melanocyte loss)	1. Storage condition of the CD4 depletion antibody may not be optimal to ensure preservation of antibody activity 2. The tumor is too large or too small on Day 12 3. The tumor was not completely removed on Day 12	1. Antibody should be stored at -80˚C and handled on ice. The CD4 + T cell depletion efficiency of the antibody should be determined by FACS if in question 2. Do not use mice with tumors less than 5 mm or larger than 15 mm 3. Make sure the tumor is completely removed
50	Difficulty in sealing the tissue using coverslip due to curling at the edge	The edges of the skin curl during transfer to the well or during fixation	Avoid any curling at the edge before and during PFA fixation Put epidermis in DPBS with the surface side facing up. Aspirate off DPBS and slowly add 1.5 mL 4% PFA to the well and then incubate
51	High background signal in immunofluorescence staining image	1. The concentration of the antibody is too high 2. Insufficient washing 3. The penetration step was omitted (Methanol + 0.3% H_2_O_2_ in -20˚C)	1. Titrate the optimal antibody concentration 2. Use more DPBS and a longer washing time 3. Make sure to conduct the penetration step
51	No or low immuno-fluorescence signal detected (poor resolution)	1. Excess fixation time 2. Confocal microscope is not properly set up 3. The concentration of the antibody is too low 4. Inappropriate choice of antibody	1. Optimize the fixation time. Long fixation time can cause antigen disruption 2. Ensure that microscope settings are properly adjusted 3. Titrate the optimal antibody concentration 4. Check the host species and isotype
51	Several skin regions have melanocyte loss but no T cell infiltration	Some mice have spontaneous white patches in their tail skin and may lack melanocytes due to developmental problems	Make sure that the tail tip is black when receiving mice from the supplier
51	Skin regions display CD8 + T cell infiltration but no melanocyte loss	Mice are too young or too old. The hair cycle of the dorsal skin is in anagen	Use 8–9-week-old female mice with telogen skin. When using genetically modified strains, use age-matched female controls

## Results

In the vitiligo mouse model described in this protocol, depigmentation occurs close to the site of tumor removal first, then other dorsal regions and the tail (Fig. 1k). Skin depigmentation is typically associated with CD8 + T cell infiltration and melanocyte loss, which are pathological features in human vitiligo disease. Consequently, we describe a FACS and whole-mount IF staining method to evaluate the CD8 + T cell infiltration and melanocyte loss in the skin (Figs. 2 and 3). We use mouse tail skin for our experimental analyses since mouse tail skin is quite similar to human skin: melanocytes are located in both the hair follicle and the epidermis.

The percentage of melanocytes in the tail skin epidermis in wild-type mice is about 0.8%. On day 33 after vitiligo induction, the melanocyte percentage decreases to 0.2% (Fig. 2f). Thus, the decrease of melanocyte percentage in the epidermis can be used as evidence of vitiligo development. Even so, a lower percentage of melanocytes can also result from other factors, such as melanocyte development defects. Thus, validation for the development of vitiligo requires additional evidence, such as CD8 + T cell infiltration.

CD8 + T cells are responsible for melanocyte loss in the vitiligo mouse model (Xu et al. 2022). In wild-type mice without vitiligo induction, the percentage of CD3 + CD8 + T cells is lower than 2% among CD45 + immune cells. On day 33 after vitiligo induction, the CD3 + CD8 + T cell percentage increases to around 50% (Fig. 2c). The percentage of CD8 + T cells in this vitiligo mouse model is associated with marked melanocyte loss after vitiligo induction (Fig. 2c, f).

Another method of monitoring vitiligo development is whole-mount immunofluorescence staining. After acquiring large-scale images, we can characterize vitiligo development based on quantification of the total cell numbers for melanocytes and CD8 + T cells. Suitable software and quantification methods are presented in Steps 51 to 53 (Fig. 3c).

The total number of melanocytes in a 1 cm^2^ control tail skin epidermis piece was about 3 × 10^4^. On day 33 after vitiligo induction, the melanocyte number decreased to fewer than 1 × 10^4^ cells. Before vitiligo induction, very few (if any) CD8 + T cells were detected in the epidermis. On day 33 after vitiligo induction, the number of CD8 + T cells increased to nearly 4 × 10^4^ in a 1 cm^2^ skin piece (Xu et al. 2022). Thus, the method presented here supports the evaluation of vitiligo development based on whole-mount IF staining with quantification of both melanocytes and CD8 + T cells.

Intriguingly, using whole-mount IF staining, we found that CD8 + T cells aggregate into small clone-like clusters rather than being evenly distributed; the loss of melanocytes also corresponds to the region where CD8 + T cells aggregate, which expands with induction time (Fig. 3d). Quantification showed the overall cluster size increases continuously with time; on Day 33, the number of CD8 + T cells in each cluster reached more than 2^10^ cells in nearly half of the clusters (Fig. 3e).

Dermal fibroblasts are known to function as regulators of skin homeostasis and to influence the pathogenesis of dermal diseases. To support investigations of biomolecules in dermal fibroblast functions, we introduce a lentivirus-mediated shRNA knockdown method for dermal fibroblasts in vivo (Fig. 4a-b). Whole-mount imaging is beneficial for side-by-side comparative analyses in the epidermis and dermis. Results from the experiment with shRNA knockdown in dermal fibroblasts can be used to infer whether a gene-of-interest plays role in CD8 + T cell infiltration, melanocyte loss, and/or vitiligo development. For example, in the mouse with fibroblasts-expressing shRNA against *Stat1*, the distribution of CD8 + T cells negatively correlated with the infected fibroblasts (Fig. 4c), and the melanocytes were positively correlated with the infected fibroblasts (Fig. 4d).

## Discussion

Vitiligo is a complex disease. Not only are there different kinds of intrinsic and extrinsic risk factors, but these factors also vary among different patients. Genome-wide association studies (GWAS) have identified more than 50 genetic risk variants for vitiligo, including genes related to cytolysis (*GZMB*, *CTLA4*, *FOXP3*) (Birlea et al. 2011; Jin et al. 2016, 2010) and melanocyte function (*TYR*, *MC1R*, XBP*1*) (Jin et al. 2012, 2007; Ren et al. 2009). Psychological stress has been reported as a precursor of vitiligo occurrence (Silverberg and Silverberg, 2015). Chronic exposure to specific chemicals like para-phenylenediamine (PPD) and monobenzyl ether of hydroquinone (MBEH) can induce skin depigmentation (Harris, 2017); there are also reports of sporadic cases of patients developing vitiligo after virus infection (Kumar et al. 2016; Philips et al. 2012; Pichler et al. 2005; Seckin et al. 2004). Due to the complex nature of how vitiligo is triggered in different patients, it is unlikely that there is a method to induce vitiligo in mouse model that could recapitulate the disease initiation process in all or majority of vitiligo patients.

Although vitiligo patients vary in how their diseases are induced, they all share three key pathological features: a. skin depigmentation; b. loss of epidermal melanocytes; c. skin infiltration of immune cells, in particular auto-reactive CD8 + T cells targeting melanocyte. Based on these clinical hallmarks, the vitiligo mouse model described here is suitable to study the cellular and molecular mechanism driving vitiligo progression. It should be noted that this vitiligo model requires transient depletion of CD4 + T cells in the early stage of induction, which not only depletes Treg cells but also other CD4 + T cells, such as Th1 cells. Thus, this model is not suitable to study the function of CD4 + T cells during vitiligo pathogenesis.

## Conclusions

This study describes a mouse vitiligo model that induces epidermal depigmentation by the combination of B16F10 inoculation and anti-CD4 antibody administration. Before the depigmentation phenotype occurs, endogenous melanocyte-specific cytotoxic CD8 + T cells have been activated, which leads to melanocyte loss. With the facility of FACS and wholemount staining technique, researchers can easily evaluate the vitiligo efficiency at 1 month post induction. In addition, there is a method to manipulate gene of interests in fibroblasts. In summary, this vitiligo model would be beneficial for studying the molecular mechanisms of vitiligo disease and testing responses to drugs.

## Data Availability

All data generated during this study are included in this article. Requests for materials should be addressed to the corresponding author.

## Abbreviations

Tregs: Regulatory T cells
MART-1: Melanoma antigen recognized by T-cells 1
TYR: Tyrosinase
IFN-γ: Interferon γ
shRNA: Short hairpin RNA
FITC: Fluorescein isothiocyanate
APC: Allophycocyanin
PE: Phycoerythrin
FOXP3: Forkhead box P3
Dct: Dopachrome tautomerase
IgG: Immunoglobulin G
EDTA: Ethylenediaminetetraacetic acid
FBS: Fetal bovine serum
DAPI: 4',6-Diamidino-2-phenylindole
DMSO: Dimethyl sulfoxide
PFA: Paraformaldehyde
DPBS: Dulbecco′s phosphate buffered saline
BSA: Bovine serum albumin
CFU: Colony-forming unit
FACS: Fluorescence-activated cell sorting
IF staining: Immunofluorescent staining
rpm: Revolutions per minute
Stat1: Signal transducer and activator of transcription 1
GWAS: Genome-wide association studies
GZMB: Granzyme B
CTLA4: Cytotoxic T-lymphocyte associated protein 4
MC1R: Melanocortin 1 receptor
XBP1: X-box binding protein 1
PPD: Para-phenylenediamine
MBEH: Monobenzyl ether of hydroquinone
